# Emergent Valve Repair for Severe Traumatic Acute Tricuspid Regurgitation in Polytrauma Patients: A Case Report

**DOI:** 10.2174/011573403X385402250709053830

**Published:** 2025-07-14

**Authors:** José Manuel Martínez Cereijo, Laura Reija Lopez, Beatriz Acuña Pais, Belén Adrio Nazar, Javier Garcia Carro, Jose Andrés Donoso Mera, Souhayla Souaf Khalafi, Angel Fernández González

**Affiliations:** 1 Department of Cardiac Surgery, Santiago de Compostela University Hospital, Santiago de Compostela, Spain

**Keywords:** Tricuspid valve repair, blunt chest trauma, emergent cardiac surgery, valve repair, polytrauma, hemodynamic stability

## Abstract

**Introduction:**

Traumatic tricuspid regurgitation resulting from blunt chest trauma is a rare complication, and the surgical options remain unclear.

**Case Presentation:**

We describe the case of a 19-year-old male who sustained polytrauma in a scooter accident.

Concomitant medical findings included massive right hemothorax, splenic burst with active bleeding, hemoperitoneum, L1-L3 spinous apophysis fracture, and 2 rib arches.

Despite 6 hours of medical treatment, including high doses of noradrenaline and dobutamine, complete stabilization was not achieved. A transesophageal echocardiogram was performed, which revealed tricuspid valve damage, with one leaflet flaring due to rupture of the papillary muscle (impression of the anterior leaflet). An emergent surgery was performed, and the valve was repaired with excellent outcomes. Additionally, hemodynamic stability was achieved post-surgery, and the repair proved to be effective and durable in the medium term.

**Conclusion:**

Severe traumatic acute tricuspid regurgitation in polytrauma patients can lead to cardiogenic shock and may require emergent correction of the valvopathy, as demonstrated in this case. In instances of traumatic acute tricuspid regurgitation caused by papillary muscle rupture, valve repair is a feasible approach and has shown favorable outcomes at midterm follow-up. Given the complex medical conditions associated with polytrauma and the typically young age of these patients, particular emphasis was placed on preserving the native valve.

## 1 INTRODUCTION

Traumatic tricuspid valve regurgitation (TTR) was first reported by Williams in 1829, with the first surgical correction performed by Cooley in the late 1950s [1, 2]. TTR of traumatic etiology is rare, occurring in only 0.02% of traumatic injuries; however, it may be underdiagnosed [3, 4]. Blunt chest trauma is the most common mechanism of injury in these cases [3].

Indications for early surgery include severe torrential regurgitation, associated with clinical and echocardiographic evidence of right ventricular strain. Repairing the valve is preferable; however, replacement of the valve is a feasible option if repair is not possible. Delay in surgical management can lead to right ventricular failure, making repair less feasible [5, 6].

Concomitant injuries are common in these cases and can influence both the decision to treat and the timing of surgery. The patient's other injuries should be carefully considered [6].

In this report, we describe the case of a 19-year-old male with polytrauma following a scooter accident. The patient required additional surgical procedures, including an emergent splenectomy and hemothorax drainage. Persistent hemodynamic instability, attributed to tricuspid valve incompetence after other causes were ruled out, necessitated emergent cardiac surgery. Valve repair was successfully performed, resulting in a favorable postoperative cardiovascular course. This represents a rare situation in the literature, as surgery for traumatic tricuspid regurgitation is often delayed in most cases.

## 2 CASE PRESENTATION

A 19-year-old man was involved in a scooter accident, colliding with a car and sustaining a severe blow primarily to the thorax. He was reanimated “*in situ*” with advanced resuscitation, which included orotracheal intubation. He was then transferred to a nearby primary hospital, where a full-body CT scan was performed. The CT revealed a massive right hemothorax, splenic burst with active bleeding, hemoperitoneum, L1-L3 spinous apophysis fracture, and 2 rib arches. Urgent laboratory tests showed a hemoglobin level of 3.4 g/dL and a hematocrit of 10%.

Emergent splenectomy was performed, and the patient was then referred to our tertiary hospital. Hemodynamic parameters showed severe instability (oliguria and MAP 50–60 mmHg despite NA at 20 mL/h and transfusion 7+9 RBCP and 2 FFP). Right chest drainage with evacuation of hemothorax and initial partial stabilization was achieved, and the patient was admitted to the ICU. After 6 hours of medical treatment, complete stabilization was not reached despite increasing high doses of noradrenaline and dobutamine, so a transesophageal echocardiogram was performed. It described a non-dilated, non-hypertrophic left ventricle (IVS 10 mm) with preserved systolic function; slight flattening of the IVS toward the left ventricle due to volume overload of the right cavities; a dilated right ventricle (baseline end-diastolic diameter 50 mm) with borderline contractility; TAPSE 17 mm; and tricuspid valve flaring of one leaflet due to rupture of the papillary muscle (impression of anterior leaflet). Free tricuspid insufficiency was present with a triangular doppler curve. Other parameters were within the normal range. Preoperative TEE images are shown in Figs. (**1** and **2**).

With these echocardiographic findings and after a lengthy deliberation about whether urgent surgery was indicated or counterproductive, we finally decided that urgent valve surgery was necessary because we considered it would not be possible to recover from right cardiogenic shock without correcting the TR.

## 3 SURGERY

A full median sternotomy was performed. Surprisingly, the pericardium was found to be ruptured on the right side, attached only to the right phrenic nerve along its course. This rupture caused herniation of the right atrium into the pleural space. The neo-diaphragm of the pericardium produced a lesion in the right atrial wall, which ruptured definitively as soon as it was touched during cannulation. This appears to have caused the right hemothorax, and the patient was likely on the verge of complete exsanguination because of it. The right atrial wall was easily repaired using a 4-0 polypropylene suture and a Teflon patch. A cardiopulmonary bypass circuit was established by cannulating the aorta, superior vena cava, and inferior vena cava. Cardiac arrest was not performed to facilitate CPB weaning. After opening the right atrium, the tricuspid valve was inspected, as shown in Figs. (**3** and **4**). A complete tear of the anterior papillary muscle was observed, with a striking avulsion of the right ventricular endocardium.

A special effort was made to repair the valve with the following considerations in mind: the postoperative course would likely be stormy, and the patient would require additional concomitant procedures. Implanting a mechanical prosthesis in such a young patient implies exposing the patient to an elevated risk of thromboembolic episodes, given his long life expectancy as well as the risks derived from anticoagulation. On the other hand, a biological prosthesis also implies the need for repeated operations due to structural valve deterioration. Clearly, the early age of the patient weighs heavily on this decision.

The valve was repaired according to the technique described in Carpentier’s *Reconstructive Valve Surgery* book. The torn portion of the muscle, along with the remnant of the subvalvular apparatus, was trimmed. An incision was made on the right side of the interventricular septum, and the fibrous tissue of the papillary muscle was fixed with two 4-0 polypropylene sutures reinforced with Teflon pledgets. A tricuspid ring (Adams Tri-Ad, size 32) was then implanted and secured with simple 2-0 coated polyester sutures. A saline test was performed, yielding a suboptimal result. A prolapse of segment A1 was observed, and a 5-0 Gore-Tex suture was placed, leading to partial improvement. A second saline test revealed a coaptation defect between the anterior and posterior leaflets, which was corrected using two 5-0 polypropylene sutures to close the commissure. After these maneuvers, the saline (water) test showed a good result, and the right atrial wall was closed with a simple 5-0 polypropylene suture. Cardiopulmonary bypass weaning was successful on the first attempt. The remaining steps were carried out in standard fashion. The echocardiographic result of the valve repair is shown in Figs. (**5** and **6**). Notably, the patient was weaned off bypass without inotropic support, strongly suggesting that the tricuspid regurgitation was the main cause of his cardiogenic shock. The rest of the postoperative course was favorable from a cardiovascular standpoint; however, the patient required an additional five days of intensive care due to his other injuries.

Once in the hospitalization unit, a follow-up echocardiogram was performed, which showed progression of tricuspid insufficiency to a moderate degree, without any clinical repercussions or impact on right ventricular function. The patient was discharged after 26 days of hospitalization. The hospital stay was prolonged due to a left pneumopericardium effusion, which required the creation of a pericardial window *via* left mini-thoracotomy. He also developed an infection with multi-resistant organisms (*Pseudomonas stutzeri* and *Staphylococcus haemolyticus*), which required treatment with intravenous antibiotics.

Due to the central venous catheter used for intravenous treatment, the patient developed a vena cava thrombosis, which led to a pulmonary embolism three months after surgery, following the interruption of anticoagulant therapy. The embolism was well tolerated, and an echocardiogram performed at that time again showed moderate tricuspid regurgitation, with no progression in severity despite the embolic event. Right ventricular function remained preserved. This serves as strong evidence that the degree of tricuspid regurgitation remained stable at the three-month follow-up.

## 4 DISCUSSION

The real prevalence of traumatic tricuspid regurgitation is probably underestimated since chronic isolated tricuspid insufficiency is usually well tolerated, and most patients experience few or no symptoms after trauma [7]. In the setting of subacute or chronic TR, the treatment is managed according to the guidelines on valvular heart disease [8].

In the literature, chordal rupture appears to be the most common cause of traumatic tricuspid regurgitation. Anterior chordal rupture is the most frequent mechanism of regurgitation, occurring in 41.9% (31/74 patients) of the cases. The other causes of traumatic tricuspid regurgitation reported in the literature are papillary muscle rupture in 27.0% (20/74 patients) and leaflet rupture in 14.8% (11/74 patients) of the cases [9, 10].

The exact mechanism of traumatic tricuspid rupture after blunt chest trauma is not known. Probably, valve rupture is secondary to compression of the heart between the sternum and the spine, resulting from a sudden increase in right ventricular pressure. Its vulnerability is compounded by the increased hydrostatic pressure during the isovolumetric contraction phase, causing avulsion of the tricuspid leaflets. The lesion is most likely to occur during the isometric systolic phase, when the heart is fully contracted and the valves close [11].

A diagnosis may be missed in the acute phase due to coexisting multisystem injuries and the subtlety of physical signs. In this case, the diagnostic challenge was significant. The patient experienced cardiopulmonary arrest and required advanced resuscitation. Hemoglobin dropped to a critical level of 3 g/dL. The massive hemothorax and acute splenic rupture were the main contributors to the patient’s condition.

Clinically overt heart failure has traditionally been the primary indication for surgery [12]. However, early surgery is recommended to prevent irreversible right ventricular dysfunction and dilatation. Van Son *et al*. noted that the only patient in their case series who did not develop right ventricular dysfunction was operated on within one month of injury [9]. It is postulated that if surgery is delayed, the papillary muscles, chordae tendineae, and involved leaflets may become atrophied, precluding successful valve repair [13]. Ma *et al*. noted this in a case series of 13 patients [13]. In one case published by Eranki *et al*, there was no evidence of right heart failure, so due to the patient's concomitant injuries, they opted to delay surgery to facilitate recovery [14]. However, this is not always an option, as exemplified by the case two described in this series.

The most extreme urgent scenario is illustrated by our case. The patient was referred to our center, still in cardiogenic shock after correction of the non-cardiac treatable causes. When persistent hemodynamic instability was noted, a transesophageal echocardiogram was performed, revealing massive tricuspid regurgitation due to papillary muscle rupture. It was difficult to determine whether the cardiogenic shock was secondary to this and to decide whether emergent cardiac surgery would be life-saving or potentially harmful for the patient.

It should be noted that if the operation is delayed, valve repair becomes more challenging due to the development of fibrosis in the valve and subvalvular apparatus [9]. Other causes of cardiogenic shock were excluded, such as active non-thoracic bleeding or neurological lesions. Considering the TEE description (referred to in the case presentation), finally, we decided to perform the surgery, preferably without cardiac arrest.

It would be desirable for the key points guiding the decision algorithm on when to treat this condition emergently to be clearer, so we can contribute to the publication of this case. It is clear when to treat once the acute phase has been resolved: in the presence of right ventricular dysfunction. Nevertheless, when should emergency surgery be performed in a polytrauma patient with concomitant injuries and cardiogenic shock not attributable to those injuries? That is the key question. It seems that we made the right decision, judging by the clinical postoperative course.

## CONCLUSION

Severe traumatic acute tricuspid regurgitation in polytrauma patients can lead to refractory cardiogenic shock and may necessitate emergent correction of the valvopathy. The postoperative course can be favorable if tricuspid regurgitation is the cause of the cardiogenic shock. In cases of traumatic acute TR due to papillary muscle rupture, valve repair is a feasible technique with good mid-term results. Repairing the valve offers significant advantages, especially in polytrauma patients who often require concomitant and delayed surgical or invasive procedures, facilitating patient recovery. Therefore, a special effort should be made to preserve the valve.

## STUDY LIMITATIONS

This is a particular case. Some medical concomitant factors could contribute to hemodynamical instability, in addition to acute TR. This fact could be more consistent with a case series.

## Figures and Tables

**Fig. (1):**
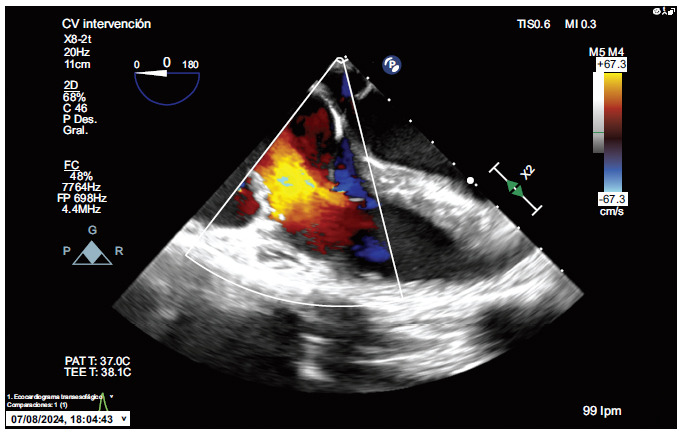
Preoperative TEE.

**Fig. (2):**
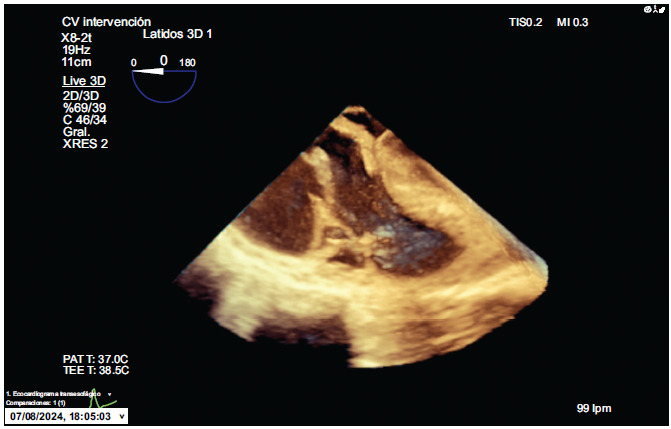
Preoperative 3D TEE.

**Fig. (3):**
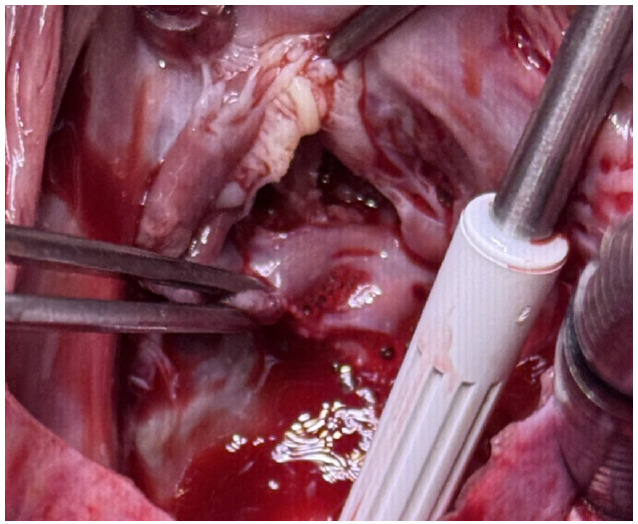
Intraoperative Image of the tricuspid valve with avulsion of the RV endocardium.

**Fig. (4):**
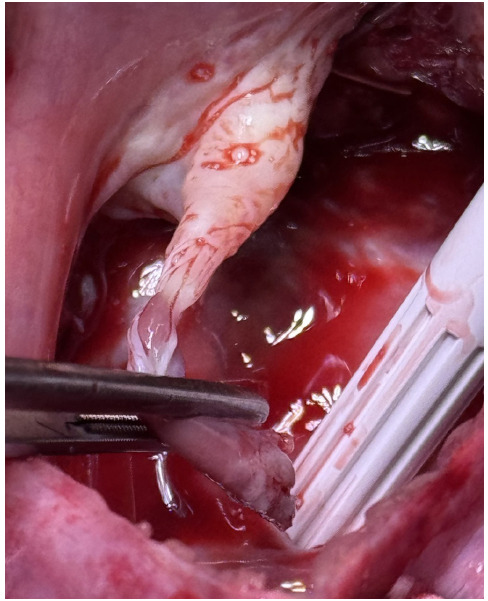
Intraoperative image of the tricuspid valve: ruptured anterior papillary muscle.

**Fig. (5):**
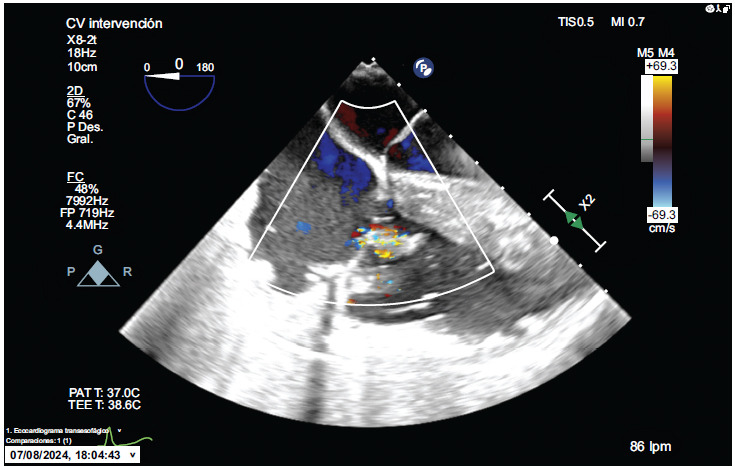
Post valve repair intraoperative TEE.

**Fig. (6):**
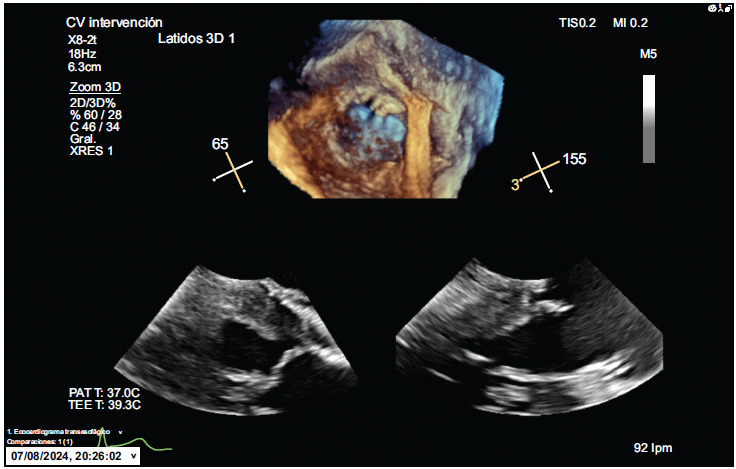
Post valve repair intraoperative 3D-TEE.

## Data Availability

The data and supportive information are available within the article.

## LIST OF ABBREVIATIONS

CPB: Cardiopulmonary Bypass
CT: Computed Tomography
FFP: Fresh Frozen Plasma
ICU: Intensive Care Unit
IVS: Interventricular Septum
LVOT: Left Ventricular Outflow Tract
MAP: Mean Arterial Pressure
NA: Noradrenaline
PSP: Pulmonary Systolic Pressure
RBCP: Red Blood Cell Package
RV: Right Ventricle
TAPSE: Systolic Displacement of the Plane of the Tricuspid Ring
TEE: Transesophageal Echocardiography
TR: Tricuspid Regurgitation
TTR: Traumatic Tricuspid Regurgitation
VTI: Velocity-Time Integral

## STANDARDS OF REPORTING

CARE guidelines and methodology were followed.
